# Supplementary material for: Future stem cell analysis: progress and challenges towards state-of-the art approaches in automated cells analysis
Source: PeerJ. 2022 Dec 21;10:e14513. doi: 10.7717/peerj.14513 (PMC9789697; doi:10.7717/peerj.14513)
Supplement: Supplemental Information 2 [file peerj-10-14513-s002.pdf]

Future Stem Cell Analysis: Progress and Challenges Towards State-of-the art Approaches in Automated Cells Analysis

Table of corrections

No.	Publisher's comment/feedback<br>Please edit the manuscript to:	Authors' actions
1.	include a rationale for why it is needed	1. A test analysis of the stem cells in the form of a microscopic image where the type of cells is rationally needs to be validated by the cells specialist (cytobiologist).<br>(Line 406 and 407).<br>2. Cell analysis has monumental purposes in assisting for accurate and fast results, which explains the rationale that the advanced technology is needed.<br>(Line 553 and 554).<br>3. Therefore, researchers realised the rationale behind the need for a system that could automatically updates the additional features without manual input, as the system can learn all possible features through deep learning.<br>(Line 613-615).<br>4. All these initiatives were rationally introduced with the priority objective to assist the experts/laboratories in automatically identifying and counting the cell without needing handcrafted analysis.<br>(Line 622-624).
2.	describe the audience it is intended for	Hence, this review study is intended for clinicians, scientists, and researchers recently enlightened about automated systems to analyse generic cell and stem cells, particularly in HSCs, iPSCs, and other cell types. This automated system could foster and empower stem cell research and its application for therapy.<br>(Line 77-80).
3.	include a Survey/Search Methodology section (see https://peerj.com/about/author-instructions/#literature-review-sections).<br>Use this section to explain how you ensured comprehensive and unbiased	**1. Search engine**<br>• In this study, we aim to understand and systemise the reviewed publications from the top list of academic search engines, which are Google Scholar and selected publisher databases with citation index traced by SCIMAGO journal ranking. For example, Elsevier, IEEE Xplore, Web of

	coverage of the literature. This must include search engines and search terms used, criteria for inclusion/exclusion of articles, etc.	Science, and several primal publishers such as JSTOR for certain manuscripts, including Dutch manuscripts, for originality and novelty concerning cell analysis approaches. (Line 92-97).<br>**2. Inclusion criteria**<br>• The publications were selected according to the inclusion and exclusion criteria of the most appropriate manuscripts from the mentioned databases. Three inclusion criterions were involved in this review, starting with the identification phase, followed by screening and inclusion phase. A total of 180 articles were selected and identified with appropriate title manuscripts contributed to cell analysis. Then, the screening phase is based on the contents of the abstract and keywords such as "microscope", "stem cell", "single-cell analysis", "cell identification", "cell counting", "image processing", "machine learning" and "deep learning".<br>(Line 106-112).<br>**3. Exclusion criteria**<br>• The review articles in the cell analysis evolutions section and the research articles without complete results were also excluded for exclusion criteria.<br>(Line 114-116).
Authors' amendment		Please take note that we have missed to declare the grant information:<br>This research was funded under Research University Grant from Universiti Kebangsaan Malaysia with grant number GUP-2019-055.
